# Supplementary material for: Axl contributes to efficient migration and invasion of melanoma cells
Source: PLoS One. 2023 Mar 29;18(3):e0283749. doi: 10.1371/journal.pone.0283749 (PMC10057740; doi:10.1371/journal.pone.0283749)
Supplement: S1 Fig — Quiesced IgR3 cells were invaded in quiescence media containing indicated concentration of FBS for 24h. Representative images are nuclei of invaded cells stained with DAPI. (DOCX) [file pone.0283749.s001.docx]

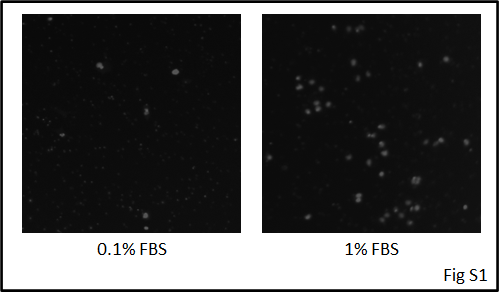


Figure S1. FBS enhances the invasion of IgR3 cells. Quiesced IgR3 cells were invaded in quiescence media containing indicated concentration of FBS for 24h. Representative images are nuclei of invaded cells stained with DAPI.
