# Supplementary material for: Axl contributes to efficient migration and invasion of melanoma cells
Source: PLoS One. 2023 Mar 29;18(3):e0283749. doi: 10.1371/journal.pone.0283749 (PMC10057740; doi:10.1371/journal.pone.0283749)
Supplement: S2 Fig — Representative images of IgR3 and WM852 cells transfected with either control or Axl siRNA and treated with or without 1 μM of R428 for 24h. Shown are random images from each well. (DOCX) [file pone.0283749.s002.docx]

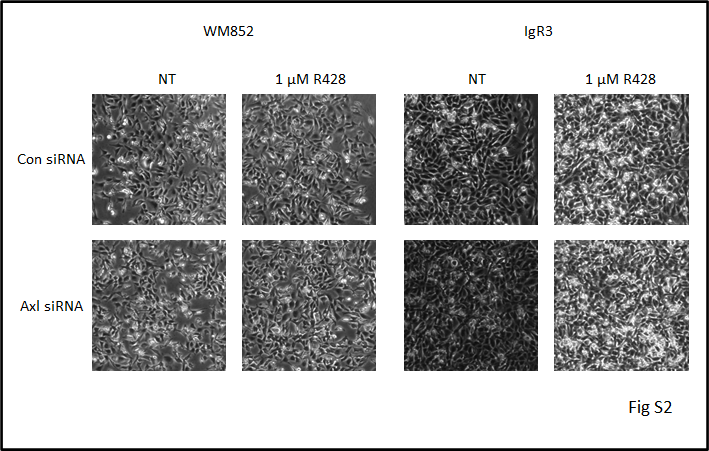


Figure S2. Low concentration (1uM) of R428 has no effect on the viability of melanoma cells. Representative images of IgR3 and WM852 cells transfected with either control or Axl siRNA and treated with or without 1 µM of R428 for 24h. Shown are random images from each well.
