# Supplementary material for: Axl contributes to efficient migration and invasion of melanoma cells
Source: PLoS One. 2023 Mar 29;18(3):e0283749. doi: 10.1371/journal.pone.0283749 (PMC10057740; doi:10.1371/journal.pone.0283749)
Supplement: S3 Fig — (A) Representative images of IgR3 and WM852 cells treated with indicated concentration of R428 for 24h. (B) Representative images of IgR3 and WM852 cells transfected with either control or Axl siRNA and treated with indicated concentration of R428 for 24h. Images were randomly taken from each well. (DOCX) [file pone.0283749.s003.docx]

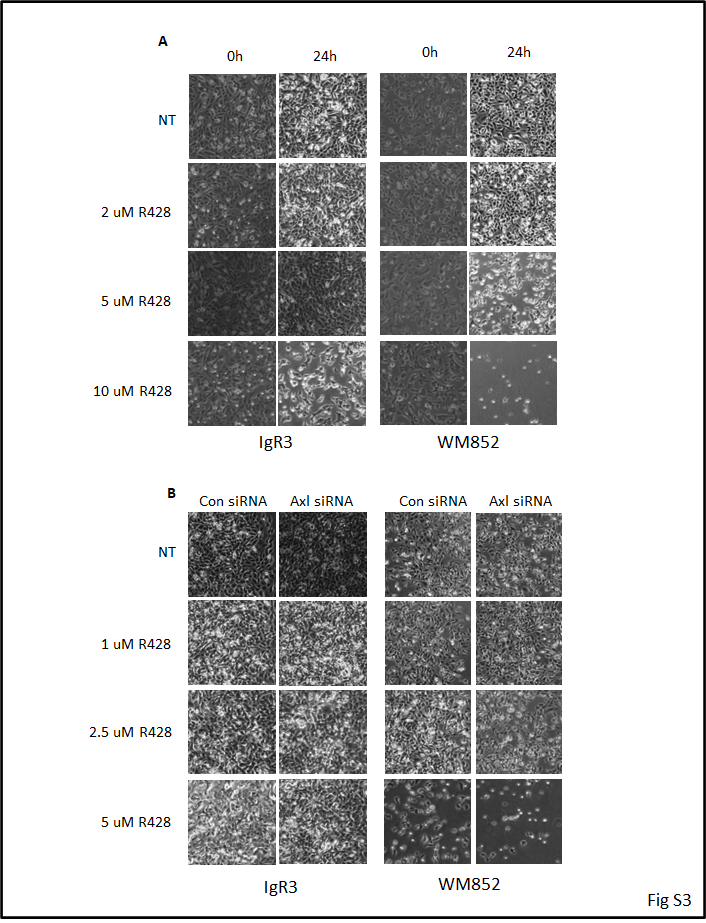


Figure S3. IgR3 and WM852 demonstrate variable tolerance to R428. (A) Representative images of IgR3 and WM852 cells treated with indicated concentration of R428 for 24h. (B) Representative images of IgR3 and WM852 cells transfected with either control or Axl siRNA and treated with indicated concentration of R428 for 24h. Images were random taken from each well.
