# Supplementary material for: Axl contributes to efficient migration and invasion of melanoma cells
Source: PLoS One. 2023 Mar 29;18(3):e0283749. doi: 10.1371/journal.pone.0283749 (PMC10057740; doi:10.1371/journal.pone.0283749)
Supplement: S1 File — (PDF) [file pone.0283749.s004.pdf]

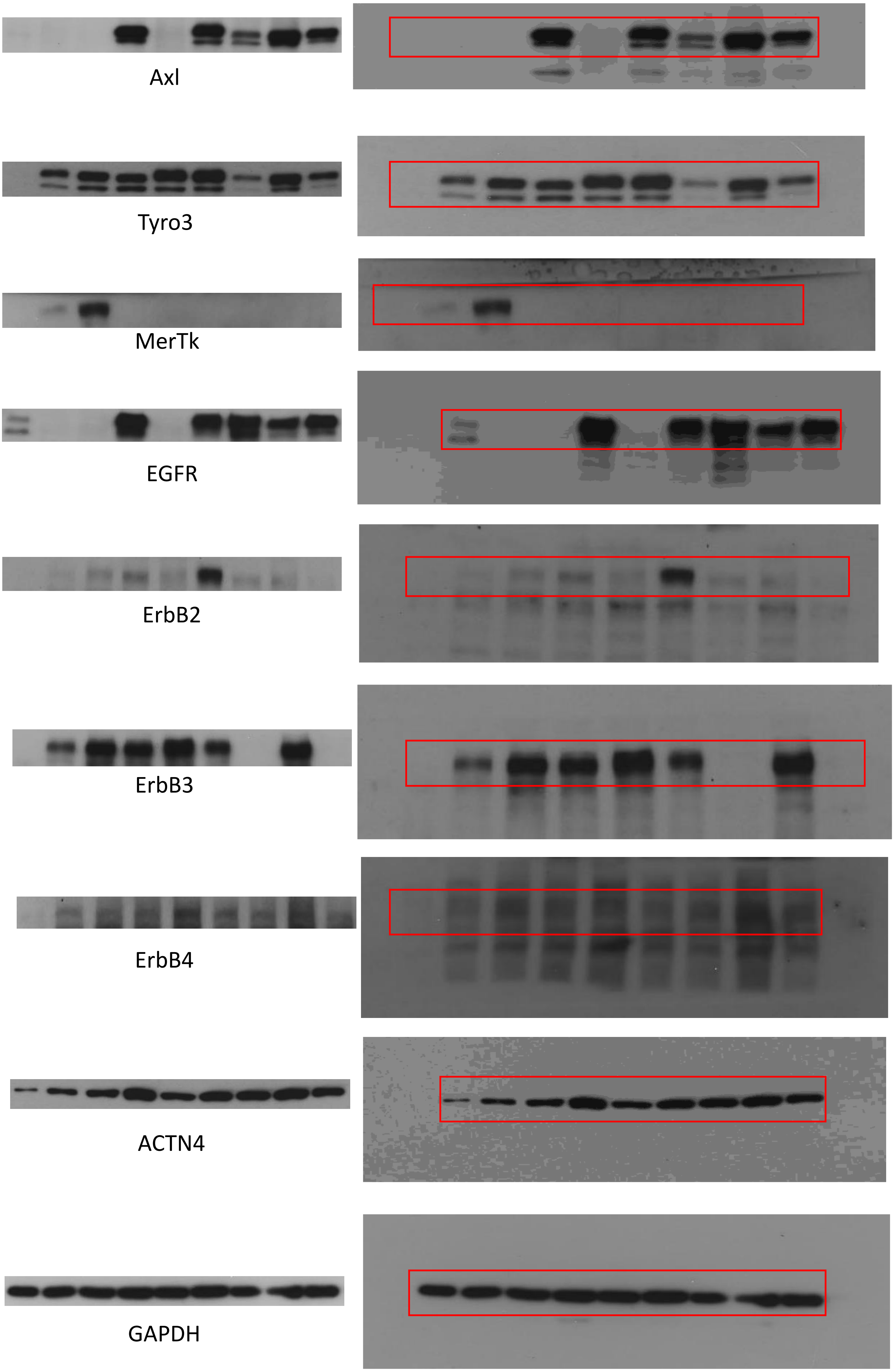

Fig. 1

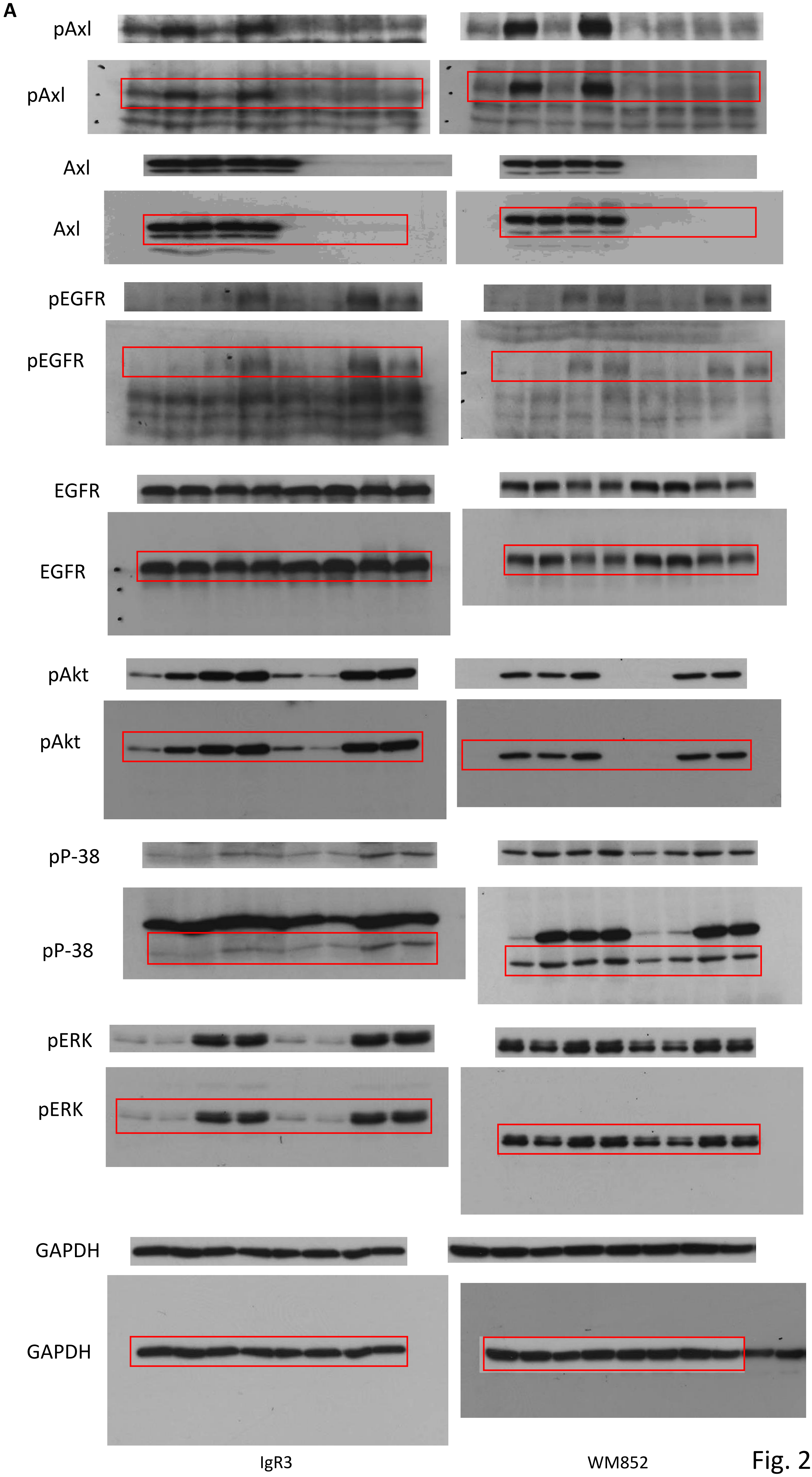
A
pAxl
pAxl
Axl
Axl
pEGFR
pEGFR
EGFR
EGFR
pAkt
pAkt
pP-38
pP-38
pERK
pERK
GAPDH
GAPDH
IgR3
WM852

Fig. 2

B

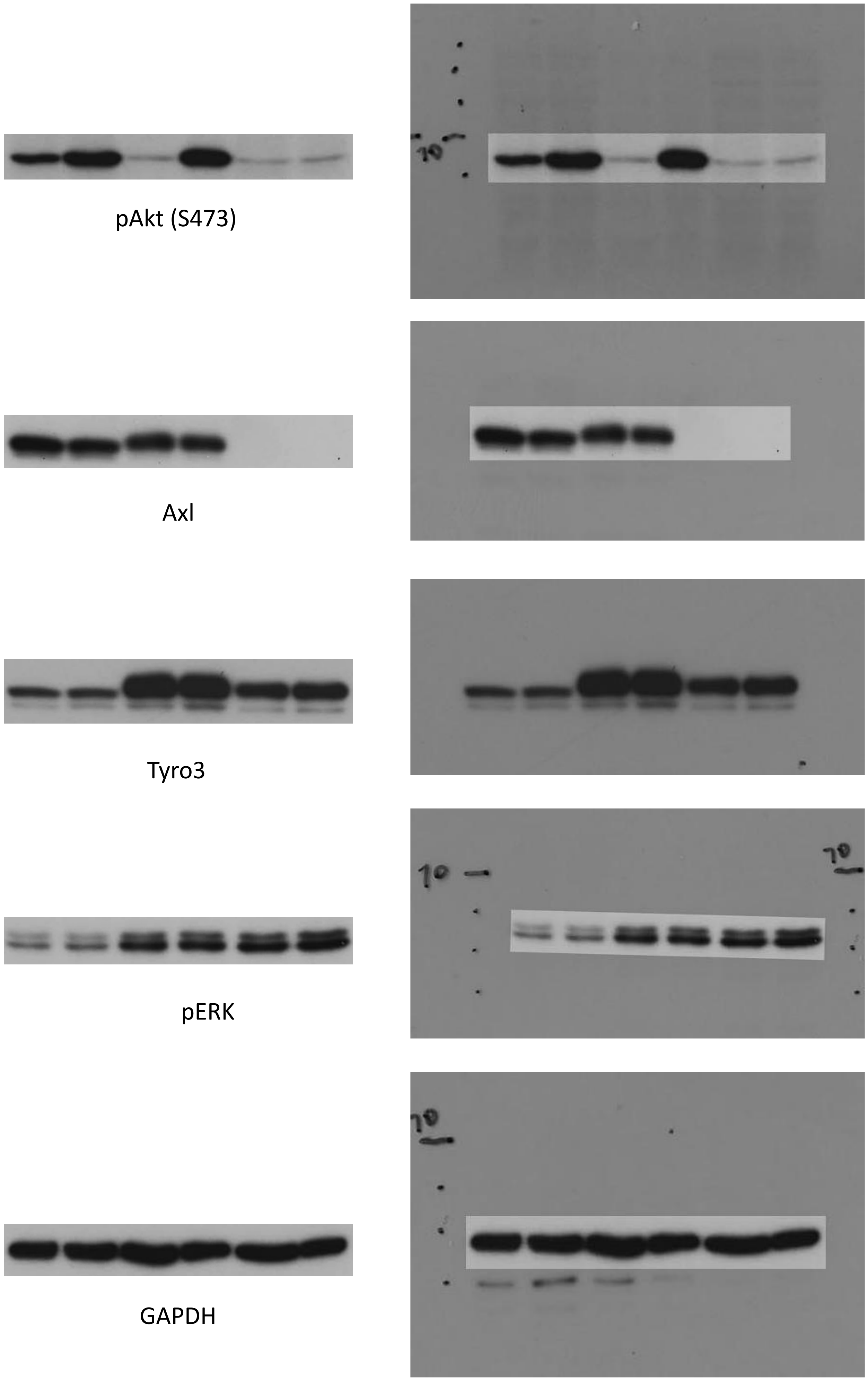

Fig. 2

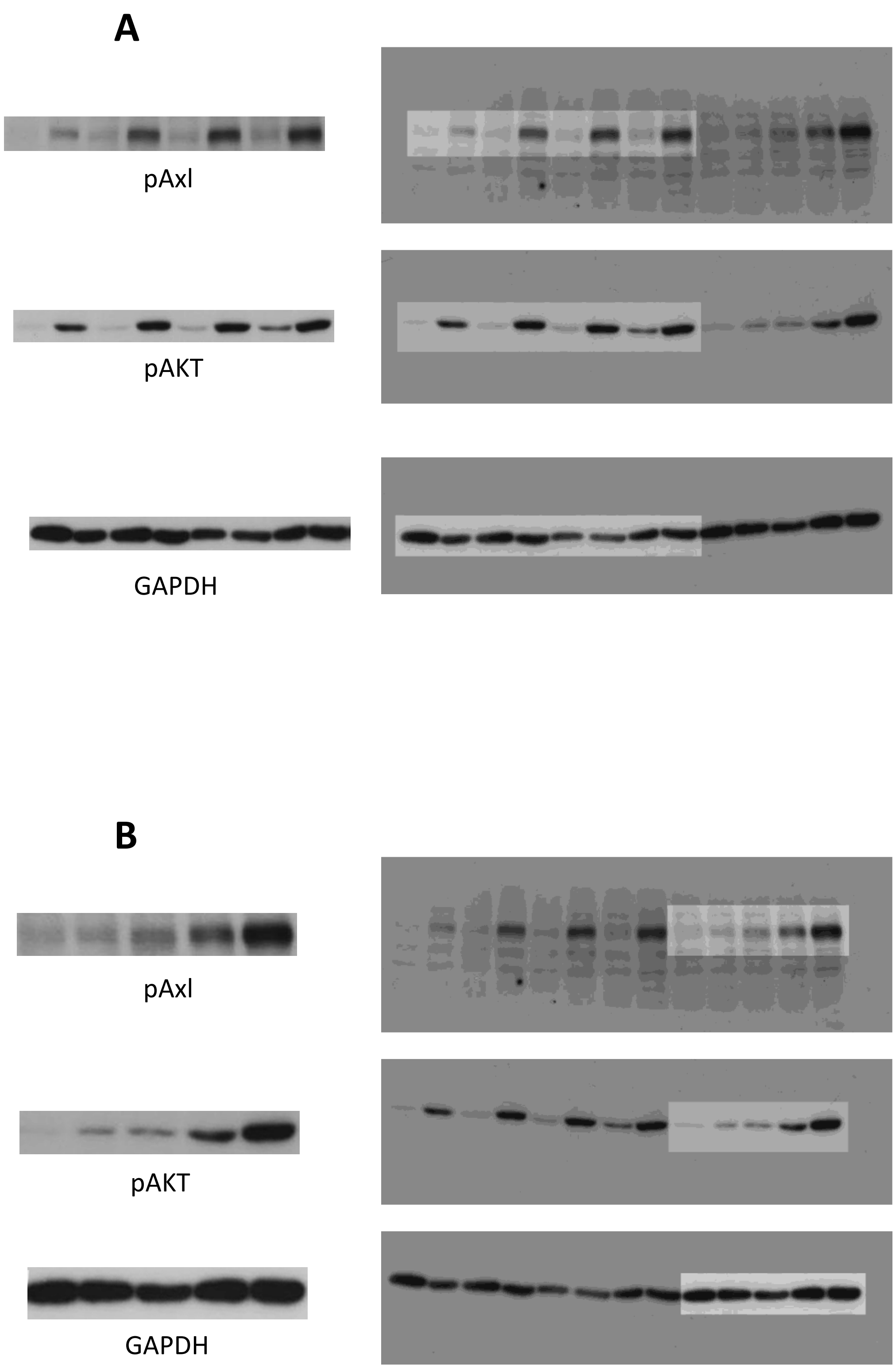

Fig. 3

A

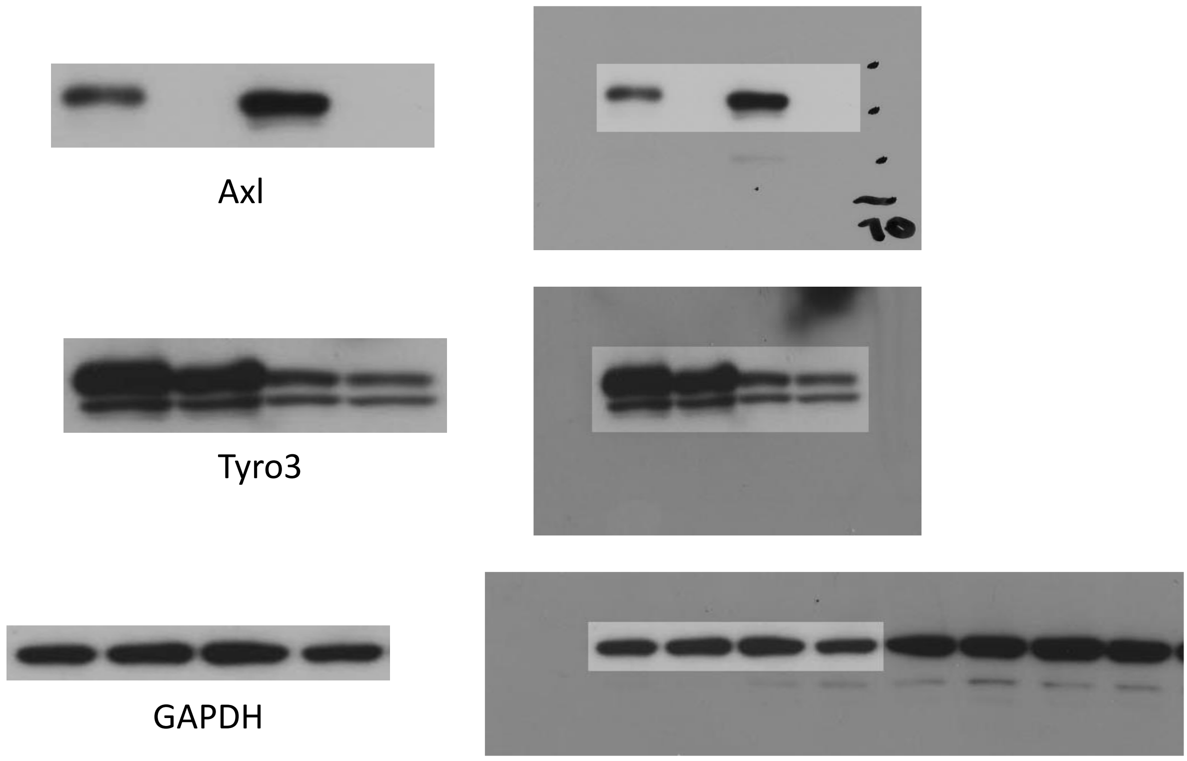

Fig. 4

A

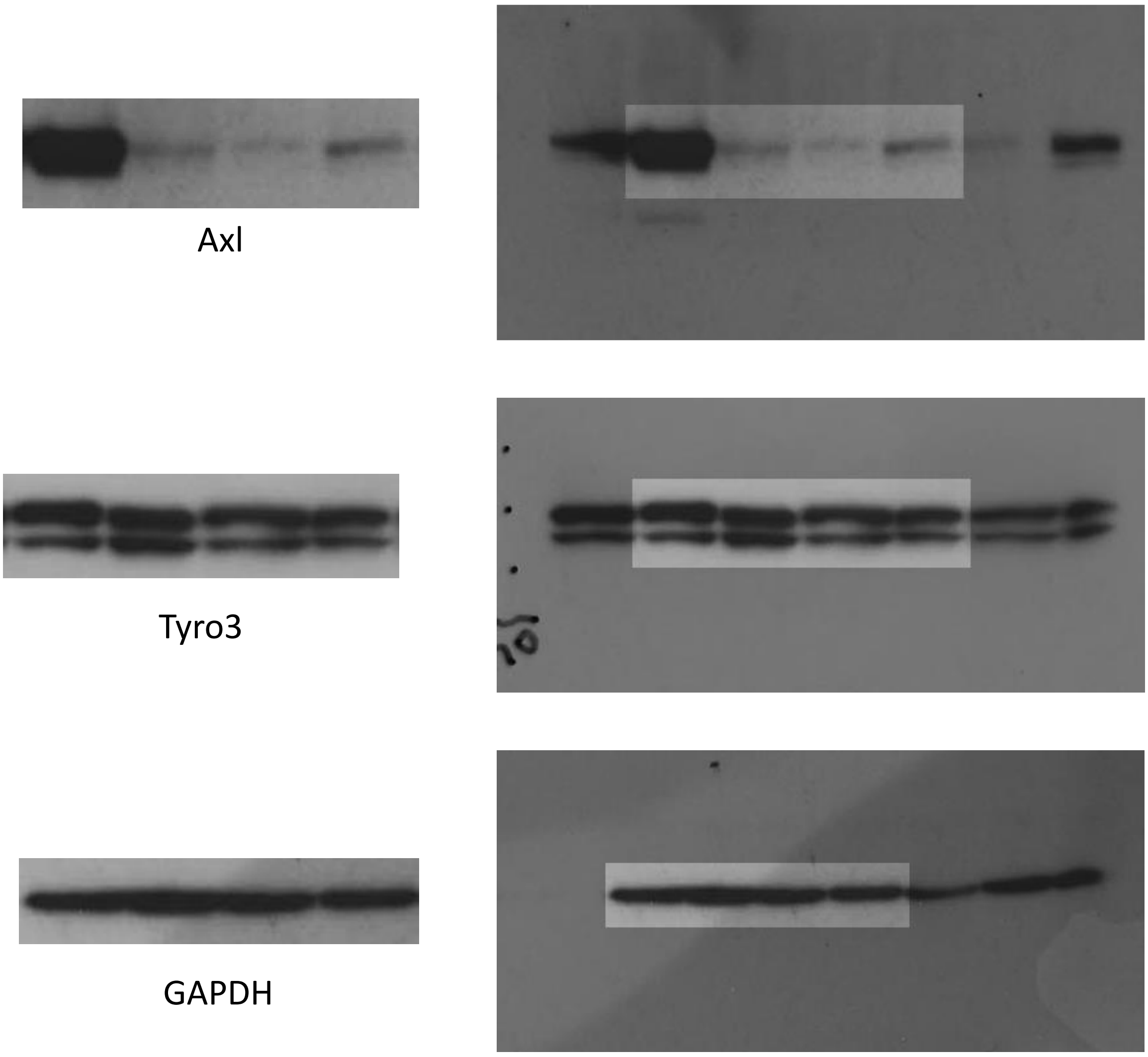

Fig. 4
